# Investigation of missense mutation-related type 1 diabetes mellitus through integrating genomic databases and bioinformatic approach

**DOI:** 10.1186/s44342-024-00005-4

**Published:** 2024-06-26

**Authors:** Dyonisa Nasirochmi Pakha, Ratih Dewi Yudhani, Lalu Muhammad Irham

**Affiliations:** 1 Department of Pharmacology, Faculty of Medicine, Universitas Sebelas Maret, Surakarta, 57126 Indonesia; 2 Faculty of Pharmacy, Universitas Ahmad Dahlan, Yogyakarta, 55166 Indonesia

**Keywords:** Bioinformatics, Genomic database, Genomic variant, T1DM

## Abstract

Though genes are already known to be responsible for type 1 diabetes mellitus (T1DM), the knowledge of missense mutation of that disease gene has still to be under covered. A genomic database and a bioinformatics-based approach are integrated in the present study in order to address this issue. Initially, nine variants associated with T1DM were retrieved from the GWAS catalogue. Different genomic algorithms such as PolyPhen2.0, SNPs and GTEx analyser programs were used to study the structural and functional effects of these mutations. Subsequently, SNPnexus was also employed to understand the effect of these mutations on the function of the expressed protein. Nine missense variants of T1DM were identified using the GWAS catalogue database. Among these nine SNPs, three were predicted to be related to the progression of T1DM disease by affecting the protein level. *TYK2* gene variants with SNP rs34536443 were thought to have a probably damaging effect. Meanwhile, both *COL4A3* and *IFIH1* genes with SNPs rs55703767 and rs35667974, respectively, might alter protein function through a possibly damaging prediction. Among the variants of the three genes, the *TYK2* gene with SNP rs34536443 had the strongest contribution in affecting the development of T1DM, with a score of 0.999. We sincerely hope that the results could be of immense importance in understanding the genetic basis of T1DM.

## Introduction

Type 1 diabetes mellitus (T1DM) is a chronic autoimmune disease marked by elevated blood glucose levels (hyperglycaemia) due to insulin deficiency. This deficiency is caused by the destruction of pancreatic islet β-cells, resulting from autoimmunity (autoimmune T1DM) and, in rare cases, there was a contribution of strong genetic factor (idiopathic T1DM) [1, 2]. Unlike type 2 diabetes mellitus (T2DM), the population of T1DM is only 10–15% among all individuals with diabetes and is commonly found at an early age, below 15 years old [1]. However, the incidence of T1DM is expected to continue to rise, with approximately 90,000 children diagnosed annually [3].

Research predominantly centres on T2DM due to its prevalence, whilst studies on T1DM remain limited. T1DM necessitates particular concern due to some challenges, including misdiagnosis, underdiagnosis, high-risk complications, and premature mortality [4]. According to Lind et al. [5], patients with T1DM had a higher risk of death from any cause, primarily cardiovascular disease, which is more than twofold compared to the general population. Moreover, this risk increases in patients with poor hyperglycaemic control. Hence, this disease continues as one of the major challenges for clinicians and researchers.

Managing T1DM mostly depends on insulin to prevent severe illness and death as well as to lower the chances of long-term macrovascular and microvascular complications [4]. Despite the presence of advanced insulins, T1DM individuals are still at a high risk of severe complications. Novel approaches are required for the prevention and treatment of T1DM [6]. Autoimmunity is a key feature of T1DM; thus, an intervention that alters the immune system could be a promising approach for treating the illness. This discovery indicates that precision medicine focusing on causative genes and the pathophysiology of complex diseases like T1DM, when fully understood, could offer more effective management [7].

The pathogenesis of T1DM involves a complex interaction between autoantibodies, genetics, and environmental factors [1]. The early autoantibodies detected in T1DM are insulin or anti-glutamic acid decarboxylase (GAD) autoantibodies. Other antibodies that can develop are protein tyrosine phosphate-related islet antigen 2 (IA-2) and zinc transporter 8 (ZnT8) autoantibodies in later stages [1, 8]. The development of these autoantibodies is related to genetic differences, age, and environmental exposure [1]. It has been documented that T1DM involves multiple gene abnormalities at different loci [8]. However, genetic risk factors alone are insufficient, with only 30% of the concordance rate of monozygotic twins having T1DM, despite long-term follow-up implicating a higher percentage. *HLA-DR3-DQ2* and *HLA-DR4-DQ8* haplotypes are the major genetic risk factors in the development of β-cell-targeting autoantibodies [9]. As a result of its association with β-cell-targeting autoantibodies, *HLA*-associated risk factors might elevate the risk of T1DM development. Furthermore, various environmental factors, such as viral infections, the initiation of food exposure, and gestational events, might cause the emergence of autoantibodies [1].

It has been documented that genome-wide association studies (GWAS) have recorded over 60 genetic factors that contributed to T1DM risk, marked by single nucleotide polymorphisms (SNPs) [10]. A study by Nyaga et al. [10] has identified T1DM-associated SNPs in regulatory networks, which are associated with the inflammation and destruction of pancreatic β-cells, the signalling of adaptive immune, and the proliferation and activation of immune cells. Although the genomic variants related to T1DM have been largely identified through the GWAS-based approach, the pathogenic variants with missense mutation are still limited to explore. Moreover, the molecular mechanism of some SNPs regions is still unknown. Hence, identifying these genetic factors at an early stage may allow for more time for prevention or treatment, as well as a slower progression of the disease [8]. This study aims to identify the genetic variants of T1DM associated with SNPs focusing on missense variants by using genomic database and bioinformatic approaches. Hence, this finding may provide an overview of potential biomarker candidates that might contribute to understanding the pathogenesis of T1DM and proposing strategies for future therapeutic approaches.

## Methods

### Retrieval of GWAS datasets

The National Human Genome Research Institute (NHGRI) GWAS Catalogue Database (https://www.ebi.ac.uk/gwas/) was used to identify the T1DM associated with SNPs [11]. Using the keyword “type 1 diabetes mellitus” (MONDO_0005147) and including the background as well as child traits data, all available data of associations were downloaded on 16 Jan 2023, resulting in 815 variant and risk alleles. Next, the data was sorted by focusing on the missense variant, odds ratio (OR) > 1, and *p*-value < 10^−8^. The missense variants are genetic variants changing the amino acid sequence of the protein. By this alteration, the pathogenic missense variant interferes with the protein function and affects the phenotypes, whilst the benign missense variants have limited impact [12]. Hence, it is essential to focus on which missense variant is thought to modify the protein function. Meanwhile, OR greater than one is considered to increase the odds among those who are exposed compared to the unexposed [13]. In addition, a *p*-value with a threshold below 10^−8^ was applied to distinguish true and false positives [14–16]. This is reasonable since multiple testing corrections were performed in GWAS that may result in a high number of false positive if the significance criterion was 0.05. Hence, using the present threshold (*p*-value < 10^−8^) improves the robustness and reproducibility of alleged associations [17].

### Projection of non-synonymous coding SNPs on protein function

After sorting and removing duplicates using Microsoft Excel, 9 SNPs were obtained. Then, those SNPs were assessed through SNPnexus (https://www.snp-nexus.org) by submitting batch queries of the 9 SNPs by selecting related annotation categories, which were PolyPhen Database and 1000 Genome Population Data. The benefits of using SNPnexus are a user-friendly interface, broad database of annotation fields, which accommodate batch searches, data visualization, and do not require substantial programming expertise nor computing resources from users. Thus, this assures that it is still applicable for analysing and interpreting sequence variants in a wide range of biological applications [18].

The PolyPhen Database aided the identification of SNP variants in affecting the protein changes in the disease, which is divided into benign, possibly damaging, and probably damaging [18–22]. This classification is based on the position-specific independent count (PSIC) scores difference between two variants (wild and mutant amino acid) [23]. The prediction of benign (range 0.0–0.49) indicates that the query substitution is likely to be benign with high confidence, whereas probably damaging (range 0.9 to 1) indicates that the query substitution is projected to be damaging with high confidence. As for the prediction of possibly damaging (range 0. 5 to 0.89) reflects that the query substitution is expected to be damaging, albeit with low confidence. Hence, this identification would estimate the effects of single amino acid substitution on protein function and structure [23, 24]. The data were extracted on 22 Jan 2023, and finally, after the whole process of analysing, this study revealed 3 SNPs that were projected to be possibly and probably damaging.

### Identification of population

The SNPnexus, also, was used to identify the population data of those SNPs using the 1000 Genome Database. The data classified the populations into 5 major continents, namely Africa, America, Europe, East Asia, and South Asia. This database allowed for a thorough description and reference of variation in human genetics; thus, helped to estimate continent-specific allele frequencies [25, 26].

### Assessing the distribution of gene expression

The three genes representing the three SNPs were then analysed to review the gene expression in various tissues using GTEx Portal (http://www.gtexportal.org/home/), extracted on 25 Jan 2023. The Genotype-Tissue Expression (GTEx) project is a large-scale resource that aids in understanding the complicated patterns of genetic variation and gene regulation found in various human tissue types. This includes analysing and interpreting the GWAS database for translation research. Thus, utilising the GTEx Portal aided in a comprehensive interpretation of this data in a variety of tissues that might be relevant to numerous diseases [27]. This study assessed the bulk tissue gene expression of each gene and focused on the top ten expressions sorted by log scale and median. The whole current method also was applied in several bioinformatics studies including chickenpox disease, systemic lupus erythematosus, and Sjogren’s syndrome [14, 16, 28].

## Results

### Screening of T1DM-associated SNPs

In this step, we identified the variants associated with T1DM using a bioinformatics-based approach. It can be seen from the data in Table 1 that nine missense variants of T1DM were identified using the GWAS catalogue database. Among these nine SNPs, three were predicted to be related to the progression of T1DM disease by affecting the protein level. Data in Table 2 depicted that *TYK2* gene variants with SNP rs34536443 were thought to have a probably damaging effect. Meanwhile, both *COL4A3* with SNPs rs55703767 and *IFIH1* genes with rs35667974 might alter protein function through a possibly damaging prediction. Among them, the *TYK2* gene with SNP rs34536443 had the strongest contribution in affecting the development of T1DM, with a score of 0.999.

**Table 1:** Missense variant of T1DM associated with SNPs with odds ratio > 1 and *p*-value < 10^−8^

**SNPs**	***p*-value**	**Odds ratio**
rs1990760	2 × 10^−14^	1.2
rs2304256	4 × 10^−9^	1.16
rs2476601	2 × 10^−111^	2
rs3184504	2 × 10^−38^	1.3
rs34536443	4 × 10^−15^	1.49
rs35667974	9 × 10^−9^	1.69
rs55703767	5 × 10^−12^	1.27
rs6043409	3 × 10^−10^	1.14
rs763361	1 × 10^−9^	1.12

**Table 2:** Genetic variants related to T1DM associated with SNPs and the effect on protein level

**SNPs**	**Chromosome**	**Variant**	**Gene**	**AA position**	**Score**	**Prediction**
rs34536443	chr19	G/C	TYK2	18	0.999	Probably damaging
				1104	0.973	Probably damaging
				919	0.973	Probably damaging
rs35667974	chr2	T/C	IFIH1	884	0.999	Probably damaging
				923	0.859	Possibly damaging
rs55703767	chr2	G/A	COL4A3	326	0.751	Possibly damaging

### The distribution of pathogenic variants of T1DM

**Fig. 1:**
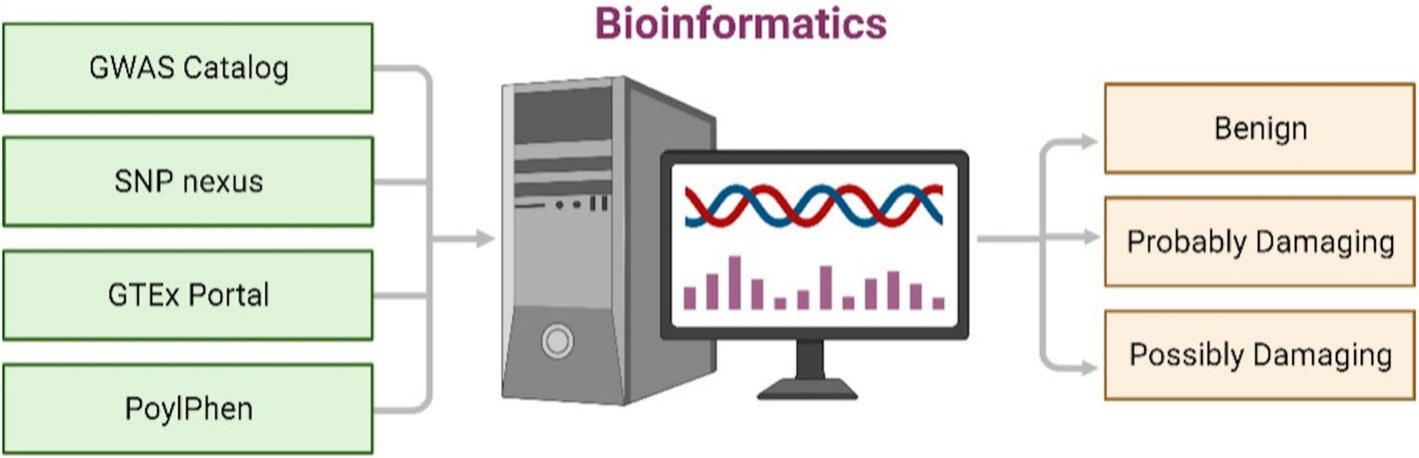
A schematic model illustrates how an integrated genomic database and bioinformatics approach can be used to identify pathogenic variants for T1DM

Table 3 illustrates the proportion of frequencies of three SNPs based on five areas using the 1000 Genome Database. Table 3 shows that rs55703767 was distributed in all populations compared to other SNPs. The highest proportion was found in the European population with the variant rs55703767 (21.17%). In contrast, the allele frequency of SNPs rs34536443 and rs35667974 was below 1% in Africa and South Asia. Meanwhile, both previous SNPs were not shown in the East Asian population (Fig. 1).

**Table 3:** The distribution of allele frequencies of three SNPs based on the region

**SNPs**	**REF allele**	**Allele**	**Allele frequency**				
		**ALT allele**	**African**	**American**	**East Asian**	**European**	**South Asian**
**rs34536443**	G	C	0.0015	0.0202	None	0.0288	0.0061
**rs35667974**	T	C	0.0008	None	None	0.0099	0.0010
**rs55703767**	G	T	0.0189	0.1542	0.1290	0.2117	0.1074

### The tissue gene expression of T1DM-associated with SNPs

**Fig. 2:**
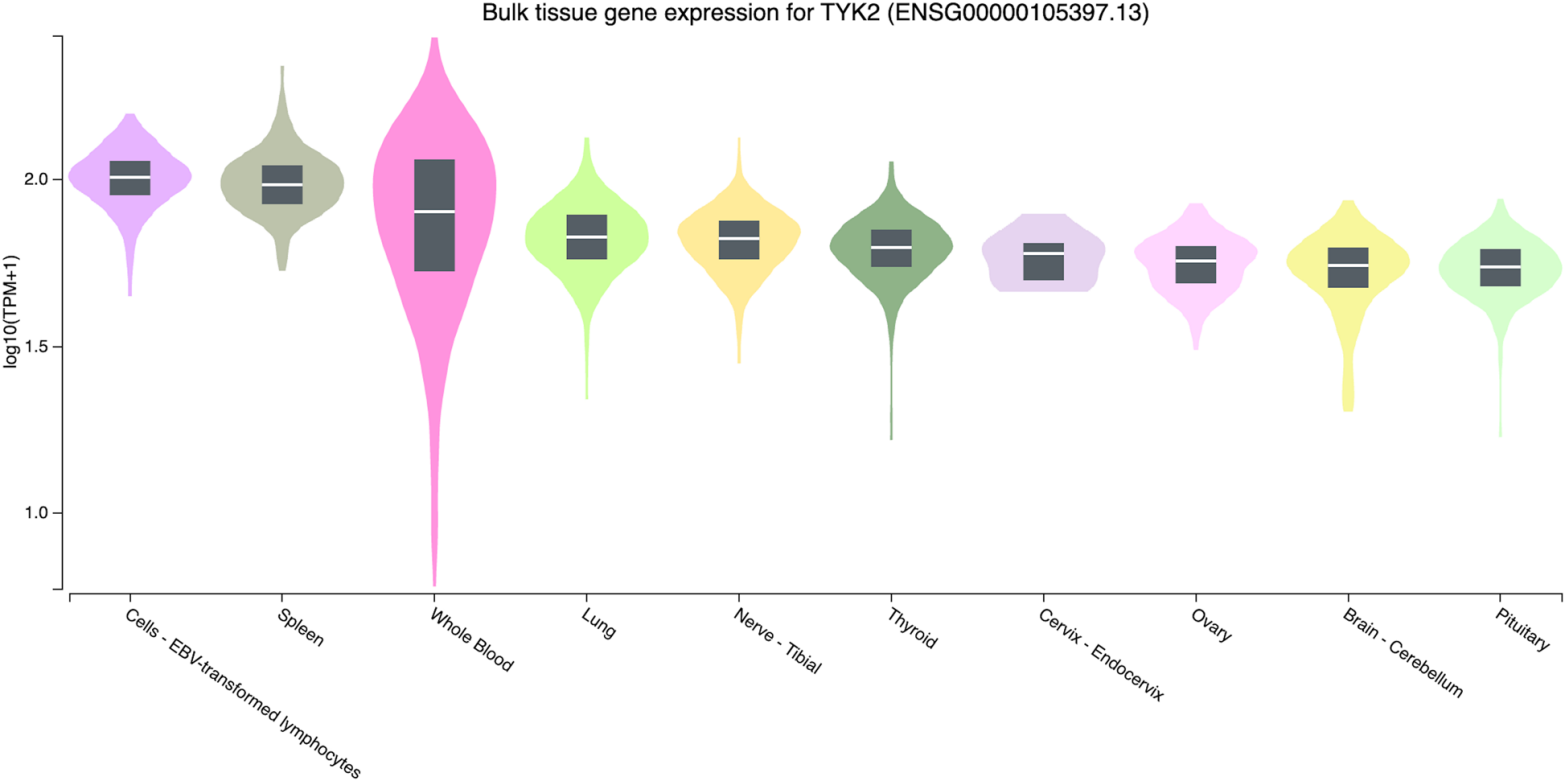
The ten most expressed tissue genes of TYK2 (based on GTEx Portal)

**Fig. 3:**
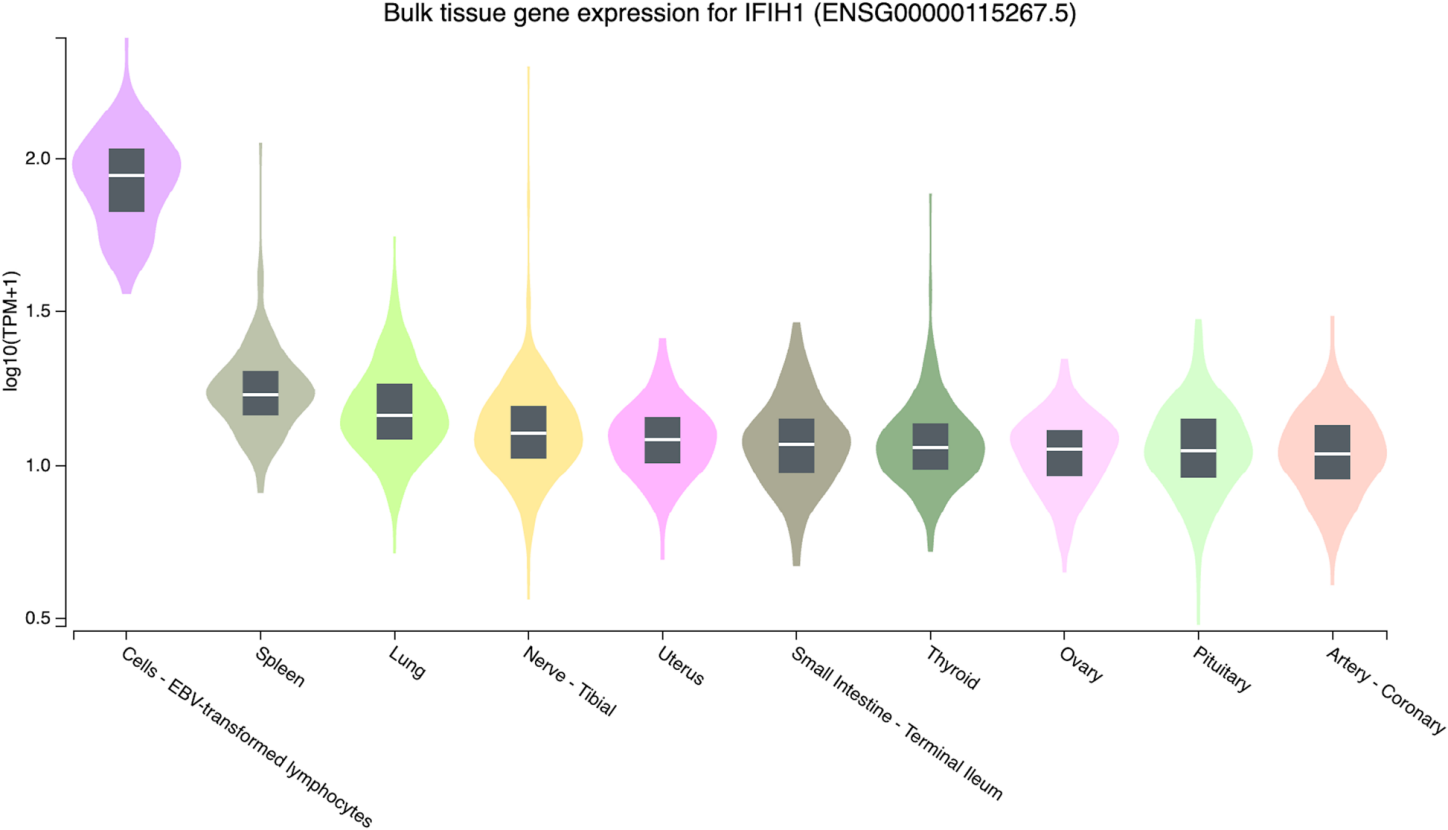
The ten most expressed tissue genes of IFIH1 (based on GTEx Portal)

**Fig. 4:**
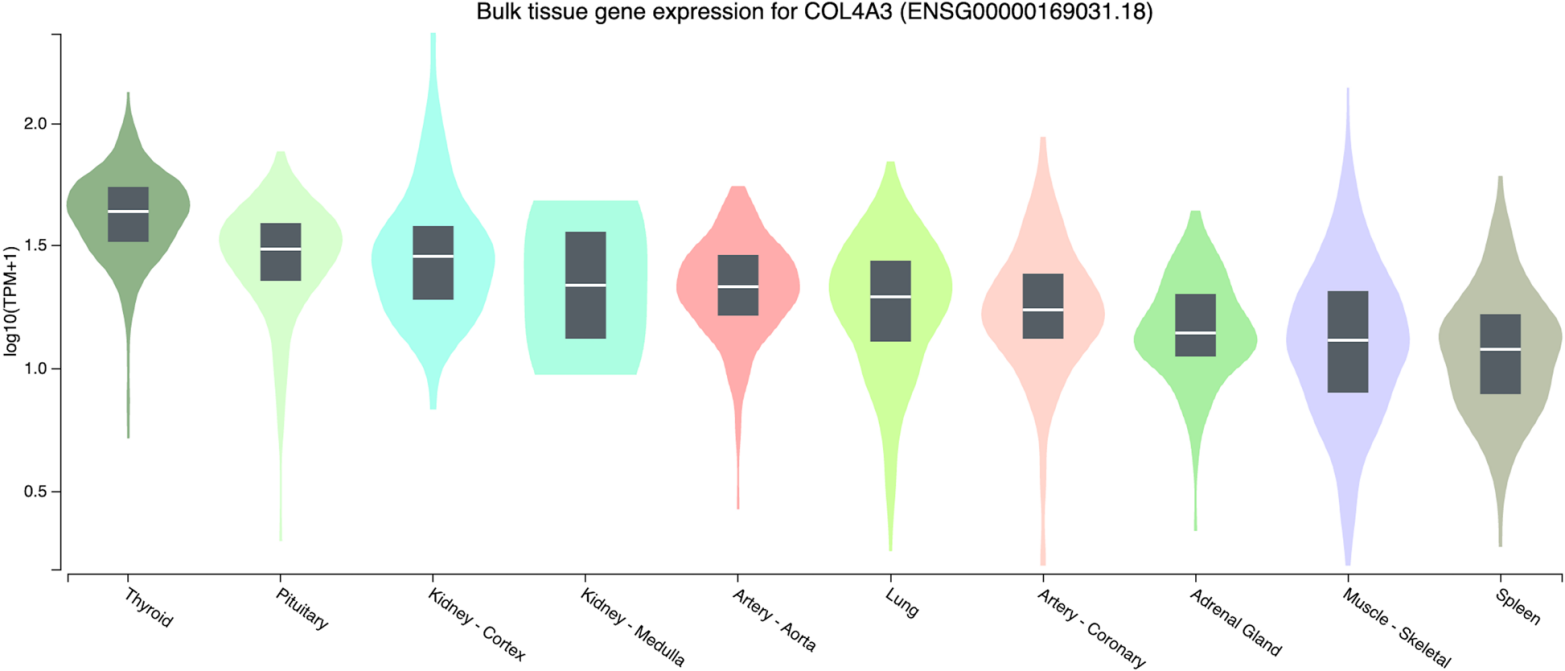
The ten most expressed tissue genes of COL4A3 (based on GTEx Portal)

GTEx Portal was used to understand the gene expression of the three genes in various tissues, presented in Figs. 2, 3 and 4. *TYK2* and *IFIH1* genes were highly expressed in cells-EBV-transformed lymphocytes and spleen (Figs. 2 and 3). Both genes were distributed higher in the lung than in other tissues. On the contrary, as shown in Fig. 4, the expression of *COL4A3* was mainly found in the thyroid, pituitary, and kidney. This study highlights that the pathogenic variants of T1DM can be identified by integrating the genomic database and bioinformatics-based approach.

## Discussion

### Variants of T1DM-associated SNPs

In this study, we used genomic databases and bioinformatics approaches to identify potential biomarkers for T1DM. interestingly, we identified that the *TYK2* gene variants with SNP rs34536443 were thought to have a probably damaging effect. It has been documented that tyrosine kinase 2 (TYK2) belongs to the family of Janus kinase (JAK), which is involved in intracellular signalling of cytokines and type I interferons (IFN-1) by phosphorylating and activating signal transducers and activators of transcription (STATs) [29]. Surprisingly, the *TYK*2 contributes to the pathogenesis of T1DM by enhancing antigen presentation via the upregulation of MHC class I and CXCL10 chemokine expression. As a result, T-cells will be activated and recruited to pancreatic islets [30]. MHC class I and CXCL10 chemokine upregulation are seen in islets from patients with T1DM. The downregulation of *TYK2* would reduce STAT1 phosphorylation and later diminish MHC class I antigen levels in haematopoietic cell lines [31]. In addition, the neutralisation of CXCL10 chemokine would suppress the emergence of T1DM in nonobese diabetic mice [32]. In line with previous studies, the inhibition of *TYK2* in mature islet cells effectively hampered the IFNα-induced MHC Class I upregulation, which led to a significant reduction of T-cell cytotoxicity [33]. Furthermore, the inhibition of *TYK2* in knock-out stem cells (KO SC)-islet models affected the endocrine percussor formation during the beginning of islet development, although it did not affect β-cells maturation and function [33]. Therefore, targeting *TYK2* through inhibition might be a promising therapeutic approach in T1DM, including preventing the development of T1DM [29].

Moreover, the loss-of-function of *TYK2* with SNPs rs34536443 has been known for the protective function against several autoimmune diseases, including T1DM, by diminishing IFN-1, IL12, and IL23 signalling [33, 34]. The minor C allele is thought to act as protection for T1DM by disrupting the activity of *TYK2* [30]. In response to type I IFN in vitro, the protective allele of rs34536443 in peripheral blood mononuclear has demonstrated reduced phosphorylation of TYK2, STAT1, and STAT2 [34]. Other studies revealed that there are impairments in IL-12 and IL23 signalling due to the impact of rs34536443, affecting a low level of pSTAT4 and pSTAT3, leading to reduced diabetogenic Th1 and Th17 populations [30, 34]. Finally, rs34536443 stands out as the only SNP with a noticeable effect on TYK2. As a result, TYK2 may be a promising target for drug-dependent inhibition in various common autoimmune disorders, including T1DM [34].

*Other variants* with SNP rs35667974 encoded the *IFIH1* gene, located at chromosome 2 with the substitution position at 884 and 923, had a contribution to the risk of T1DM and were categorised as probably damaging (score 0.999) and possibly damaging (score 0.859), respectively. Interferon gene induced by helicase C domain 1 (*IFIH1*), also known as melanoma differentiation-associated 5 (MDA-5). *IFIH1* is located on chromosome 2q24.3 and regulates the expression of an early β-responsive type I interferon (IFN) gene. This gene encodes a viral RNA-activated apoptotic protein, thought to play a role in recognising and promoting a clearance response in virus-infected cells [35]. Previous study indicates that a decreased level of expression or functioning of *IFIH1*, known as a viral RNA receptor, protects against T1DM. In T1DM susceptible individuals, both normal or activated antiviral responses could trigger apoptosis of infected pancreatic β cells, which express an elevated quantity of *IFIH1* RNA, and lead to type 1 interferon signalling, improving HLA class I expression on β-cells, thus enhancing cytotoxic CD-8 T cell-mediated destruction [36].

Viruses are shown to play a significant role in initiating the autoimmunity that contributes to the occurrence of T1DM. Among the multiple viral genotypes studied currently, enteroviruses have been consistently linked to T1DM in humans since EV showed tropism specific to the pancreas [37]. Enteroviruses (EV), also known as small non-envelope RNA viruses, have been revealed to play a crucial role in initiating an autoimmune process, leading to the destruction of the β-cells pancreas. Moreover, the EV genome has been detected in the circulation of T1DM patients [38, 39]. Other evidence indicates that EV infection is present in the β-cells of patients with fulminant diabetes [40, 41]. These underlying *IFIH1* gene polymorphisms can mediate the molecular relationship between specific virus triggers and autoimmune responses in T1DM [42].

A number of *IFIH1* polymorphisms were linked with T1DM, especially the rs1990760 (G/A) polymorphism in the United Kingdom population (substitution of an alanine for a valine in codon 946 of exon 15) being most closely linked with protection from disease development (OR = 0.86, *p* = 1.42 × 10^−10^ for the G allele) [42]. In contrast, the A allele of the rs1990760 *IFIH1* polymorphism strongly correlated with T1DM in Hungarians (OR = 1.29; *p* = 0.002) [43]. In a Russian population, two rare *IFIH1* variants, rs35744605 and rs667974, were significantly associated with a reduced incidence of T1DM [44]. The *IFIH1* gene with SNP rs35667974 (substitution isoleucine for valine in codon 923 of exon 14) also showed a protective effect against T1DM (OR = 0.43, 95% CI = 0.43–0.61, *p* = 1.3 × 10^−14^) [45].

The IFIH1 gene significantly affects the innate immune response to viral infection. Binding viral replication-derived dsRNA to IFIH1 causes immune cells to release proinflammatory cytokines. This local inflammation and activation of antiviral defence mechanisms are designed to eliminate infection and induce apoptosis in virus-infected cells. Meanwhile, this immune system malfunctions among specific genetically susceptible individuals, eliciting excessive, progressive inflammation and prolonged β-cell death, consequently predisposing them to T1DM. It provides evidence that the *IFIH1* gene is an excellent candidate gene for further investigation into T1DM [37].

Table 2 documented that the *COL4A3* gene with SNP rs55703767, located at chromosome 2 with the substitution position at 329 (G/A), took into account the risk of T1DM and was categorised as possibly damaging (score 0.751). The rs55703767 is a common missense mutation (G > T; Asp326Tyr) in exon 17 of the collagen type IV alpha 3 chain *(COL4A3)* gene. It has been documented that rs55703767 was significantly related to the protection from diabetic nephropathy, any albuminuria, combined phenotype of chronic kidney disease and diabetic nephropathy, and macroalbuminuria. Also, this SNP in *COL4A3* was more strongly associated with men than women [46].

The *COL4A3* gene encodes α3 chains of type 4 collagen, which is the major structural protein of the kidney basement membranes (BMs). Variants and mutations in the *COL4A3* gene may result in diabetic kidney disease (DKD) in young adults with maturity-onset diabetes, and pathogenic *COL4A3* mutations have been identified for Alport syndrome (AS), a progressive inherited nephropathy [47]. In addition, mutations in *COL4A3* have been reported in patients with focal segmental glomerulosclerosis, a condition in which scar tissue progresses on the glomeruli, which might result in proteinuria and renal failure [46]. Type IV collagen is typically an extracellular structural protein that forms a collagen branch network, a crucial component of BMs [48]. Abnormal collagen homopolymers might result in molecular folding, secretion, and extracellular matrix formation. Misfolded proteins can be secreted into the BM or accumulate in podocytes, altering the glomerular selective barrier structure and initiating downstream pathological pathways [47]. Moreover, dysregulation of interactions between cells, collagen IV basement membrane, cell adhesion, proliferation, survival, and differentiation have been linked to several pathologic illnesses, including chronic kidney disease [46].

T1DM is associated with a lengthy onset of nephropathy. Initially, the patient exhibits hyperfiltration, characterised by high glomerular filtration rate values, about twice as much as the normal value, and occasional episodes of microalbuminuria. Diabetic nephropathy is identified by glomerular hypertrophy and the thickening of basement, tubular, and glomerular membranes. Additionally, the extracellular matrix accumulation in glomerular membranes was also profound. These pathogenic structures ultimately result in tubulointerstitial and glomerular fibrosis and sclerosis [49]. Multiple mechanisms are involved in the onset and progression of diabetic nephropathy, including the interaction between hyperglycaemia-induced metabolic and hemodynamic alterations and genetic predisposition, thereby establishing the stage for kidney injury [49]. The most significant risk factors for the emergence of diabetic nephropathy are hyperglycaemia, high blood pressure, and genetic predispositions [50]. Therefore, along with the increasing cases of diabetes, diabetic nephropathy is the most common factor causing end-stage renal failure [49]. Based on these, a missense variant in *COL4A3* (rs55703767) was associated with DKD, and *COL4A3* mutations have been implicated in basement membrane diseases such as familial FSGS and Alport symport. Therefore, this risk allele may contribute to the aetiology of diabetic kidney disease, making it a great candidate for target therapy.

Differing from T1DM, neither study up to this current research has identified a connection between variants of *TYK2* and *COL4A3* genes and the likelihood of developing gestational diabetes. Unlike the two genes studied earlier, Kochenborger et al. [51] reported that *IFIH1* gene expression was inversely correlated to HbA1c levels. It indicated a connection between variants of the *IFIH1* gene and its protective effect on the likelihood of developing gestational diabetes mellitus (GDM). Meanwhile, in T2DM, the *TYK2* promoter variant was linked to an increased incidence of diabetes and associated with dysfunctional insulin production [52, 53], whereas the T allele of *COL4A3* was associated with T2DM with a protective role [54]. Most studies have shown that *IFIH1* with rs35667974 protects against the onset of T1DM, but the link with T2DM is unknown and requires further research. However, this differs from *IFIH1* with rs1990760, which demonstrated a lower incidence of T2DM in the Iraqi population [55, 56].

Genome-wide association studies (GWAS) have found a link between genetic differences in single nucleotide polymorphisms (SNPs) and a higher risk of both T2DM and GDM in different groups of people, including polymorphisms at the transcription factor 7-like 2 gene (*TCF7L2*), adiponectin gene (*ADIPOQ*), and fat mass and obesity-associated gene (*FTO*). The relationship has been suggested due to the shared pathophysiological processes of insulin resistance and chronic inflammation in both GDM and T2DM [57]. Hence, to understand the association between each gene in this study with T2DM and GDM, further studies need to be done to validate the association.

### The distribution of pathogenic variants of T1DM-associated with SNPs

This study displayed that Europeans had the highest distribution of the three SNPs related to T1DM rather than other populations. Based on the DIAMOND project, initiated by WHO in 1990, populations from China and South America had the lowest incidence, which was < 1/100,000 per year, whilst the highest one was noticed in Sardinia, Finland, Sweden, Norway, Portugal, UK, Canada, and New Zealand [58, 59].

Based on Table 3, the American also had a higher distributed SNP, except for SNP rs35667974. Both rs35667974 and rs34536443 alleles were below 1% in African and South Asian, and those SNPs were not found in the East Asia population. The SEARCH project in the United States reported that the incidence and prevalence rates of T1DM in the youth were higher in non-Hispanic whites (2.0/1000 and 23.6/100,000, respectively) than in other ethnicities [59, 60]. Additionally, according to a meta-analysis by Mobasseri et al. [61], the incidence of T1DM in Africa, Asia, Europe, and America was statistically significant, with 8/100,000, 15/100,000, 15/100,000, and 20/100,000 population, respectively. Although the prevalence of T1DM was not significant in Africa at 3.5/10,000, the rates in Asia, Europe, and America were statistically significant, with 6.9/10,000, 12.2/10,000, and 12.2/10,000 people, respectively. Hence, the incidence and prevalence of T1DM are increasing worldwide [61].

Furthermore, the rs55703767 were widely distributed in the five populations compared to the other alleles (Table 3). This SNP has been known as one of the loci associated with DKD and has the strongest signal compared to other loci associated with DKD. The rs55703767 is a common missense variant in exon 17 of *COL4A3*. This variant’s risk allele impacted the risk of macroalbuminuria or end-stage renal disease, and its frequency was prevalent among Europeans (20%) and East Asians (13%), but modest in Africans (2%) [62]. This distribution supported the findings in this study, which displayed 21.17% in Europe, 12.9% in East Asia, and 1.89% in Africa (Table 3). Mutations and variants of *COL4A3* contribute to Alport syndrome and DKD [46]. Interestingly, around 40% of those with T1DM have an increased risk of DKD. Individuals with DKD are at a higher risk of developing cardiovascular disease and premature mortality [63].

### The tissue gene expression of T1DM-associated with SNPs

Transcriptional changes in diverse tissues and organs are predominantly responsible for and reflective of tissue and organ dysfunction. In a significant part, transcriptional variation mediates causal links between genotype and complex traits. Thus, insights into tissue-specific gene expression (TSGE) profiles can contribute to a better understanding of the aetiology of disease [64]. We utilised the Genotype-Tissue Expression (GTEx) portal database at http://www.gtexportal.org/home/ for determining gene expression levels in human tissues of the genetic variation linked to T1DM. The GTEx is an established gene and its associated tissues database for studying the relationship between genetic variation and gene expression, as well as other molecular phenotypes in multiple reference tissues. It is also beneficial for elucidating the intricate patterns of genetic variation and gene regulation throughout various types of human tissue [65].

The GTEx database documented that the ten most tissues-specific expressions of *the TYK2* and *IFIH1* genes were similar. Both genes were highly expressed in cells-EBV-transformed lymphocytes, spleen, and lung (Figs. 2 and 3). Specifically, *TYK2* was also highly expressed in whole blood. Figure 2 correlates with Gencards’ database, showing that *TYK2* is predominantly expressed in spleen and blood cell components such as peripheral blood mononuclear cells (PBMC), B lymphocytes, and CD4 and CD8 T cells [66]. The high level of *TYK2* expression in those tissues might be due to its role in immunity and cytokine signalling mechanisms. Poelzl et al. [67] documented that *TYK2* regulates cytokine signalling and promotes immunity against viral and bacterial infections in humans and mice. In comparison, the *IFIH1* protein is expressed in a tissue-specific manner, including the lung and spleen, according to UniPort (https://www.uniprot.org/uniprotkb/Q8R5F7/entry) [68]. This conforms to the depiction in Fig. 3. The spleen contains the three main types of mononuclear phagocytes: macrophages, dendritic cells, and monocytes. They identify pathogens and cellular stress, eliminate dying cells and foreign substances, control tissue homeostasis and inflammatory responses, and contribute to adaptive immunity [69].

In contrast to the former genes, the expression of *COL4A3* was primarily found in the thyroid, pituitary, and kidney (Fig. 4). Similarly, with the HPA dataset, *COL4A3* was primarily expressed in the kidney and thyroid gland. Also, based on the dataset, the lung was in the top three of the expression of *COL4A3* in organs [70]. However, this was slightly different from the data from GTExPortal (Fig. 4), although the lung was still in the top ten of most expressed tissue gen of *COL4A3*. It has been documented that *COL4A3* had a strong signal related to a thinner glomerular basement membrane and protection against albuminuria and diabetic kidney disease [71]. Hence, this might explain the expression in the kidney.

This bioinformatics approach has identified three risk genes associated with T1DM that may provide an understanding of the T1DM pathogenesis and assist in identifying potential therapeutic targets. Although GWAS have provided new insights for assessing the association between SNPs and disease statistically, limitations still emerge. One of the limitations is that GWAS only account for a modest proportion of the missing heritability, including T1DM, which has complex traits. Additionally, current genome-wide complex trait analyses indicate that SNPs may account for one-third to two-thirds of the heritability of the majority of complex traits. One possible explanation is due the SNPs fall short of the strict significance level; thus, these SNPs with a minor effect may be ignored [72]. Furthermore, the clinical predictive value of GWAS is limited. Thus, screening population for the genetics is not practicable due to the highly exceed number of false positives compared to true positives [72, 73].

Although the current study did not cross-validate with any additional databases other than those stated, it was assessed using three large datasets, namely GWAS, SNPnexus, and GTEx Portal. Thus, this study can provide information about the missense genetic variations related to T1DM disease, which may aid in the identification of novel treatment targets. However, considering the limitations, further preclinical and clinical studies with larger sample sizes and adequate study designs are necessary to validate these findings.

These further studies may be helpful for analysing more complex interactions with phenotypes and complex diseases, as well as translating these discoveries into clinical practice. This includes analysing a druggable target that is related to the pathophysiology and genetics of T1DM. Following that, identifying and comprehending the disease’s heterogeneity can contribute to tailored therapy that improves treatment success, resulting in a better long-term outcome for individuals with T1DM [74].

## Summary

This present bioinformatics study displayed that the missense genetic variants of *TYK2*, *IFIH1*, and *COL4A3* genes were associated with T1DM disease. The loss-of-function of *TYK2* rs34536443 and decreased level of *IFIH1* are known for the protective function of T1DM. Similarly, the *COL4A3* rs55703767 is significantly related to protection from diabetic nephropathy. Therefore, these genes are excellent candidates for further investigation that may provide new insights into the pathogenesis and therapeutic target in T1DM.
